# Development of a Patient-Derived 3D Immuno-Oncology Platform to Potentiate Immunotherapy Responses in Ascites-Derived Circulating Tumor Cells

**DOI:** 10.3390/cancers15164128

**Published:** 2023-08-16

**Authors:** Thomas J. Gerton, Allen Green, Marco Campisi, Minyue Chen, Iliana Gjeci, Navin Mahadevan, Catherine A. A. Lee, Ranjan Mishra, Ha V. Vo, Koji Haratani, Ze-Hua Li, Kathleen T. Hasselblatt, Bryanna Testino, Trevor Connor, Christine G. Lian, Kevin M. Elias, Patrick Lizotte, Elena V. Ivanova, David A. Barbie, Daniela M. Dinulescu

**Affiliations:** 1 Division of Women’s and Perinatal Pathology, Department of Pathology, Brigham and Women’s Hospital, Harvard Medical School, Boston, MA 02115, USA; 2 Department of Medical Oncology, Dana-Farber Cancer Institute, Boston, MA 02215, USA; 3 Belfer Center for Applied Cancer Science, Dana-Farber Cancer Institute, Boston, MA 02215, USA; 4 Division of Dermatopathology, Department of Pathology, Brigham and Women’s Hospital, Harvard Medical School, Boston, MA 02115, USA; 5 Whitehead Institute for Biomedical Research, Cambridge, MA 02142, USA; 6 Department of Obstetrics, Gynecology, and Reproductive Biology, Brigham and Women’s Hospital, Harvard Medical School, Boston, MA 02115, USA; 7 Division of Gynecologic Oncology, Dana-Farber Cancer Institute, Boston, MA 02215, USA

**Keywords:** PDOTS, ovarian cancer, ascites, epigenetic, methylation, ICB, PD-L1, PD-1

## Abstract

**Simple Summary:**

The clinical implementation of novel precision medicine strategies in high-grade serous ovarian cancer (HGSOC), the most common and aggressive ovarian cancer subtype, are urgently needed. Targeted immunotherapeutic combinations that maximize drug benefits are of particular interest. Unlike lung cancer and melanoma, immunotherapeutic responses using immune checkpoint blockade (ICB) in high-grade serous ovarian cancer have been lower than expected and longer-term remissions are uncommon. Evidence now demonstrates that global DNA hypermethylation plays a critical role in immune evasion. Consequently, epigenetic reprogramming strategies could be beneficial in potentiating immunotherapeutic responses by reversing tumor escape mechanisms and enhancing immune cell activation. The current study details the development of ex vivo 3D patient-derived platforms for rapid testing of immunotherapeutic combinations in high-grade serous ovarian tumor metastases and tumor ascites. It further proposes the implementation of epigenetic adjuvants to potentiate systemic ICB responses and eradicate circulating tumor cells responsible for wide, aggressive metastases in this poor prognostic disease.

**Abstract:**

High-grade serous ovarian cancer (HGSOC) is responsible for the majority of gynecology cancer-related deaths. Patients in remission often relapse with more aggressive forms of disease within 2 years post-treatment. Alternative immuno-oncology (IO) strategies, such as immune checkpoint blockade (ICB) targeting the PD-(L)1 signaling axis, have proven inefficient so far. Our aim is to utilize epigenetic modulators to maximize the benefit of personalized IO combinations in ex vivo 3D patient-derived platforms and in vivo syngeneic models. Using patient-derived tumor ascites, we optimized an ex vivo 3D screening platform (PDOTS), which employs autologous immune cells and circulating ascites-derived tumor cells, to rapidly test personalized IO combinations. Most importantly, patient responses to platinum chemotherapy and poly-ADP ribose polymerase inhibitors in 3D platforms recapitulate clinical responses. Furthermore, similar to clinical trial results, responses to ICB in PDOTS tend to be low and positively correlated with the frequency of CD3+ immune cells and EPCAM+/PD-L1+ tumor cells. Thus, the greatest response observed with anti-PD-1/anti-PD-L1 immunotherapy alone is seen in patient-derived HGSOC ascites, which present with high levels of systemic CD3+ and PD-L1+ expression in immune and tumor cells, respectively. In addition, priming with epigenetic adjuvants greatly potentiates ICB in ex vivo 3D testing platforms and in vivo tumor models. We further find that epigenetic priming induces increased tumor secretion of several key cytokines known to augment T and NK cell activation and cytotoxicity, including IL-6, IP-10 (CXCL10), KC (CXCL1), and RANTES (CCL5). Moreover, epigenetic priming alone and in combination with ICB immunotherapy in patient-derived PDOTS induces rapid upregulation of CD69, a reliable early activation of immune markers in both CD4+ and CD8+ T cells. Consequently, this functional precision medicine approach could rapidly identify personalized therapeutic combinations able to potentiate ICB, which is a great advantage, especially given the current clinical difficulty of testing a high number of potential combinations in patients.

## 1. Introduction

High-grade serous ovarian cancer (HGSOC) represents more than 70% of all epithelial ovarian cancers and is responsible for the vast majority of gynecologic-related deaths [1,2,3]. Despite an initially robust clinical response to platinum-based chemotherapy, HGSOC has a high mortality rate, as patients often relapse within 2 years following diagnosis [4,5,6,7]. Patients with *BRCA*-mutated HGSOC receive additional treatment in the form of poly-ADP ribose polymerase inhibitors (PARPi), but regularly develop resistance to these therapies as well [8]. Unfortunately, there are no long-term viable therapeutic options for HGSOC patients with intrinsic or acquired platinum resistance, although some responses have recently been seen with a folate receptor alpha-targeted antibody-drug conjugate (ADC) [4,5,6,7,9]. Furthermore, despite impressive immuno-oncology (IO) advances in lung, head and neck cancers, and melanomas, several limitations remain. Multiple tumor subtypes, including HGSOC, are notoriously resistant to immunotherapy. Multiple clinical trials of ovarian cancer patients receiving immune checkpoint blockade alone (ICB) and in various combinations are currently being evaluated. To date, clinical trial response rates seen in HGSOC patients receiving immune checkpoint blockade have been limited (<15%) [10]. Current research in the field has shifted towards maximizing ICB efficacy through the use of combination treatments [11]. These combinations include PARPis, anti-angiogenic therapies, cytokine therapy, and chemotherapy [12,13]. Although these results are more promising compared to immunotherapy alone, there is still an urgent need to develop accurate testing platforms and ICB response biomarkers for use in clinical trials. 

Several ICB inhibitors against cytotoxic T-lymphocyte-associated protein 4 (CTLA-4), programmed cell death protein 1 (PD-1), and/or programmed death-ligand 1 (PD-L1) have been approved by the FDA for the treatment of metastatic melanoma, advanced kidney cancer, metastatic non-small cell lung cancer, and unresectable or metastatic triple negative breast cancer [9,14,15]. These approved monoclonal antibodies (mAbs) for ICB therapy include ipilimumab (αCTLA-4), nivolumab (αPD-1), pembrolizumab (αPD-1), and atezolizumab (αPD-L1) [16,17]. A high tumor mutation burden and increased PD-L1 expression in cancer cells have often been associated with effective ICB responses and favorable prognosis in multiple tumor subtypes [18,19]. The presence of CD8+ T cells inside the tumor or at the tumor periphery, known as tumor-infiltrating lymphocytes (TILs), and increased PD-1 expression within T cells have also been associated with a more robust clinical response and outcome [18,19]. The current study further investigates the functionality of effector T cells in newly diagnosed HGSOC patients to better understand the low response to ICB in HGSOC clinical trials. To this end, we analyzed brisk (high) tumor-infiltrating lymphocytes (TILs) that infiltrate diffusely within the tumor and compared them to non-brisk (low) TILs that show only focal infiltration or absent TILs (no TILs or minimal TIL infiltration), respectively. We further sought to identify therapeutic strategies to potentiate immune activation and function.

Specifically, we employed epigenetic priming to reverse aberrant changes in DNA methylation, which is a key mechanism that enables tumor cells to evade the immune system, induce tolerance and develop resistance to ICB [20,21,22,23,24,25,26,27,28,29,30,31,32,33,34,35,36,37,38]. Specifically, DNA methylation is known to play a key role in modulating cytotoxic T cell function and exhaustion, while decreased tumor PD-L1 expression is associated with hypermethylation [20,21,22,23,24,25,26,27,28,29,30,31,32,33,34,35,36,37,38]. Consequently, epigenetic reprogramming strategies may be beneficial in cancer immunotherapy by modulating immune cell differentiation, proliferation and function while reversing escape mechanisms employed by cancer cells [20,21,22,23,24,25,26,27,28,29,30,31,32,33,34,35,36,37,38,39]. The discovery of the ten-eleven translocase (TET) family of 5-mC hydroxylases, including TET1, TET2 and TET3, which convert 5-methylcytosine (5-mC) to 5-hydroxylmethylcytosine (5-hmC), has uncovered new layers of epigenetic modifications in cancer. Most importantly, global genome methylation levels and, specifically, changes in 5-hmC expression have been identified as a sensitive predictor for patient prognosis and therapeutic response in multiple solid tumors, including ovarian cancer [39]. Thus, global 5-hmC loss is associated with a decreased response to standard chemotherapy, shorter time to relapse and poor overall survival in patients newly diagnosed with HGSOC [39]. We have further identified a targetable pathway to reverse epigenetic 5-hmC loss, both genetically and pharmacologically [39]. Interestingly, epigenetic priming enables the rescue of 5-hmC loss, reduces the number of cancer stem cells, restores sensitivity to platinum chemotherapy, and increases overall survival in chemoresistant animal models [39]. Consequently, identifying prognostic epigenetic markers and altering therapeutic regimens to incorporate DNA methyl transferase inhibitors (DNMTi) in highly methylated tumors with poor prognosis could have important clinical implications for treatment in newly diagnosed HGSOC patients.

The current study shows that epigenetic priming potentiates ICB responses in tumor models and ex vivo 3D patient-derived platforms. Interestingly, epigenetic priming using 5-azacytidine (5-aza), a DNMTi, and givinostat, a histone deacetylase inhibitor (HDACi), prior to ICB delivery, enhances TIL infiltration and overall survival in the murine ID8 tumor model [40]. As outlined in the elegant study by Stone et al., the ID8-VEGF model is well suited and has been used extensively for ICB testing in ovarian cancer, as it is an immunocompetent model with an immunosuppressed tumor microenvironment (TME) [40]. However, it does not recapitulate HGSOC development since the ID8 parental cells are derived from the murine ovarian surface epithelium (OSE), while the vast majority of patient tumors originate in the distal fallopian tube rather than OSE [40]. More recently, novel syngeneic HGSC models, which have a fallopian tubal origin, key genetic alterations, and better recapitulate the clinical disease, have been described [41]. Consequently, additional research is needed to assess whether this potentially synergistic combination is effective in syngeneic models, which better recapitulate ICB responses [41], and in ex vivo testing platforms utilizing patient samples [42,43,44]. Our results indicate that epigenetic priming mediates the rapid secretion of key tumor-derived cytokines known to augment T and NK cell activation and cytotoxicity, including IL-6, IP-10 (CXCL10), KC (CXCL1), and RANTES (CCL5). It further upregulates the early activation of immune markers in both CD4+ and CD8+ T cells and potentiates ICB responses in ex vivo patient-derived platforms and in vivo syngeneic HGSOC models. The development of novel microfluidic devices embedded with patient-derived organotypic tumor spheroids (PDOTS) and autologous immune cells is key for the rapid IO testing of clinical samples. Here, we report the optimization of a 3D immuno-oncology PDOTS platform for ex vivo screening of HGSOC-derived ascites, which has broad implications for interrogating systemic immune and circulating tumor cell responses. The implementation of a predictive IO testing platform will allow for the rapid screening of a large number of personalized therapeutic combinations prior to their testing in patients. 

## 2. Materials and Methods

### 2.1. Digital Spatial Profiling of the Tumor-Immune Microenvironment

Digital spatial profiling (DSP) of tumor samples was performed on the GeoMx platform (NanoString, Seattle, WA, USA) [45]. Regions of interest (ROIs) were selected by a board-certified pathologist. Immunofluorescence for pan-Cytokeratin (tumor marker), CD45 (immune marker), and SYTO 13 (DNA stain) (NanoString Technologies, GMX-PRO-MORPH-HST-12) guided the selection of ROIs. Samples were incubated with 77 oligonucleotide-conjugated and photocleavable antibodies (NanoString Technologies, GMX-PROCO-NCT-HICP-12, GMX-PROMOD-NCT-HICT-12, GMX-PROMOD-NCT-HIAS-12, and GMX-PROMOD-NCT-HIODT-12) as well as negative and positive controls. After incubation and imaging, the ROIs were segmented into pan-CK+ (tumor) and pan-CK− (stroma) areas. Oligo tags were released from the areas of illumination (AOIs) via targeted exposure to ultraviolet radiation, followed by hybridization and counting using the NanoString GeoMx nCounter system [45]. The GeoMx DSP Data Analysis Suite (v2.4.0.421) was used to evaluate the raw count data output from the nCounter platform. Initial QC was performed using the default parameters. 

### 2.2. Tumor Cell Culture Assays

A2780Res and Kuramochi cells were cultured in RPMI base medium supplemented with 10% fetal bovine serum (FBS) and 1% penicillin-streptomycin. BPPNM, KPCA.A, KPCA.B, and KPCA.C cells were all cultured in a DMEM base medium supplemented with 10% FBS, 1% penicillin-streptomycin, 10 ng/mL epidermal growth factor (Sigma Aldrich, E4127-1MG), 5 mL of 100× insulin–transferrin–selenium (Fisher Scientific, 41400045, Waltham, MA, USA), and 100 ng/mL of cholera toxin (Sigma Aldrich, 227036-1MG, Burlington, MA, USA). Human ascites samples were cultured in a base medium of RPMI supplemented with 10% FBS and 1% penicillin-streptomycin.

Frozen peripheral blood mononuclear cells (PBMCs) purchased from STEMCELL (70025) were thawed and washed in 5 mL of RPMI with 10% human serum (Sigma-Aldrich, H5667), 1% penicillin-streptomycin, and 200 µM of L-Glutamine (Gibco, 25030149, Billings, MT, USA). The washed PBMCs were suspended in 1 mL of the complete medium at 37 °C for 45 min. Then, T cells were isolated using the EasySep™ Human T Cell Isolation Kit (STEMCELL, 17951, Tokyo, Japan), immediately followed by activation using the Human T Cell Activation/Expansion Kit (Miltenyi Biotec, 130-091-441, Bergisch Gladbach, Germany), according to the manufacturer’s instructions. The activated T cells were cultured in the complete medium with 100 U/mL of human IL-2 (ThermoFisher, PHC0021, Waltham, MA, USA), and the medium and IL-2 were refreshed every three days. After 14 days of activation, the T cells were used for the downstream assay by day 35.

### 2.3. Assessment of Global Methylation (5-hmC) Levels in HGSOC 

A2780Res, BPPNM, and KPCA.A cells were plated at 50,000 cells per well in a Falcon 8-well culture slide. After waiting 24 h for the cells to adhere, the medium was removed and replaced with 5-aza-treated medium. Aliquots of 5-aza (Sigma Aldrich, A2385-100MG) were thawed prior to each treatment and diluted to 1000× their final concentration in DMSO prior to a final 1:1000 dilution using the supplemented cell culture medium. Controls were treated with an equivalent volume of DMSO in supplemented cell culture medium. Treatment was readministered 48 h after initial cell plating; 72 h after initial cell plating, the cells were washed twice using 1× PBS and fixed for 30 min using 4% paraformaldehyde. After fixation, the cells were permeabilized for 15 min using 0.5% Triton X-100. Cell DNA was then denatured using 2N HCl for 30 min. The acid was then neutralized by adding 100 mM Tris-HCl (pH 8) for 10 min. All samples were then washed three times using 1× PBS supplemented with 0.6 µM EDTA. A blocking buffer consisting of 1× PBS supplemented with 5% goat serum and 0.3% Triton X-100 was then used to block the samples for 60 min. Then, 5-hmC (Active Motif, 39769, Carlsbad, CA, USA; 1:800) primary antibody dilutions consisting of 1× PBS with 1% BSA and 0.3% Triton X-100 were added to the respective samples and incubated overnight at 4 °C. The following day, the samples were washed three times with 0.1% PBS-T and incubated with an AF488 goat anti-rabbit secondary antibody (Invitrogen #A11008, 1:1000) for 60 min in the dark. After another three washes using 0.1% PBS-T, the samples were briefly dipped in deionized water to dissolve any excess salts from the PBS and DAPI was applied for nuclear staining. The samples were imaged on an EVOS FL Auto 2 microscope after adding coverslips. For quantification, three images were taken at 20× magnification and positive cells were counted using the ImageJ software (v1.53f).

### 2.4. Tumor-Derived Secreted Cytokine/Chemokine Profiling in Response to DNMTIs

KPCA.A, KPCA.B, and KPCA.C cell lines were seeded at 150,000 cells per well in a 6-well tissue culture plate and allowed to grow for 48 h. The cells were washed twice with 1× PBS and replenished with serum-free DMEM medium alone or containing 10 µM 5-aza. Azacytidine was replenished every 24 h. After 72 h, the cell culture supernatants were collected on ice, centrifuged to remove any cell debris, and then flash-frozen on dry ice. The secreted cytokines and chemokines were profiled using the Mouse Cytokine 44-Plex Discovery Assay from Eve Technologies (Calgary, AB, Canada).

### 2.5. Optimization of Patient-Derived Organotypic HGSOC Spheroids (PDOTS) in 3D Microfluidic Devices

Patient tissue studies reviewed and approved by the Institutional Review Board of Brigham and Women’s Hospital (#2006P002438) and Dana Farber Cancer Institute (#02-051) were conducted in accordance with the Declaration of Helsinki. Patient-derived tumor ascites samples were collected using paracentesis and centrifuged into a cell pellet. The supernatant was aspirated, and the pellet was resuspended in ACK lysis buffer (Thermo Fisher, A1049201). After maximum hemolysis was observed, 1× PBS was added to the sample and centrifugation was repeated to ensure removal of red blood cells and hemolytic products. The final cell pellet was slightly agitated to promote the resuspension of the cells as spheroids. The resuspended sample was then filtered through 100 μm and 40 μm filters to generate three spheroid fractions: S1 (>100 μm), S2 (40–100 μm), and S3 (<40 μm). S2 fractions containing both tumor and immune cells within the tumor-immune microenvironment were used for ex vivo cultures of solid HGSOC tumor samples, as previously described [43,44]. S2 + S3 fractions containing circulating tumor spheroids and systemic immune cells were used for ex vivo cultures of HGSOC tumor ascites samples. On ice, a mixture of 3.00 µg/mL collagen, 1× phenol red, and distilled water was adjusted to a pH of 7.3–7.4 with 0.5 N NaOH. The S2 + S3 fraction of spheroids were pelleted again using centrifugation at 300× *g* for 3 min. This pellet was resuspended in the collagen mixture and 10 µL of this spheroid-collagen mixture was loaded into an IdenTx microfluidic device (AIM Biotech, DAX-1) as previously described [43,44]. The devices were incubated at 37 °C for 35–40 min to allow the collagen to polymerize and form a matrix. After polymerization, 300 µL of RPMI medium supplemented with 10% FBS, 1% penicillin-streptomycin, and 100 U/mL IL-2 (Miltenyi Biotec, 130097746) was divided equally between the four medium ports. For treatment conditions, various concentrations of 5-aza, HDACi, PARPi (Talazoparib, AbMole Biosciences, BMN637, Houston, TX, USA), αPD-1 (Fisher Scientific, 501360845), αPD-L1 (Fisher Scientific, 501360846), and cisplatin (Patterson Vet, 07-893-4099, Loveland, CO, USA) were administered. The medium was replenished on the third day of incubation for all conditions.

For tumor spheroid–T cell co-culture studies, Kuramochi tumor cells were cultured in a 6-well ULA plate (Corning, CLS3471-24EA, Corning, NY, USA) at 500,000 cells per well for 24 h. After spheroid formation occurred, the cells were filtered through a 100 μm filter and a subsequent 40 μm filter. The 40–100 nm spheroids isolated by filtration were collected and labeled as the S2 fraction. The S2 fraction was mixed with T cells isolated from PBMCs at a ratio of 1:3 in the collagen mixture listed previously. The co-culture was then loaded into the IdenTx microfluidic devices following the above procedure, with 40,000 cells loaded into each cell port. The medium used was composed of 5% human serum, 5% FBS, 1% penicillin-streptomycin, 100 µM L-glutamine, and 6000 U/mL IL-2. Media treatment conditions were 2 µM 5-aza and 2 µM HDACi; 200 µg/mL αPD-1 and αPD-L1; and 2 µM 5-aza, 2 µM HDACi, 200 µg/mL αPD-1 and αPD-L1. Devices were stained for live–dead analysis after 3 days of culture.

### 2.6. Immunofluorescent Imaging of PDOTS

After approximately 24 h in an untreated device, the medium was drained from the wells and replaced with a 1:100 dilution of FcR block (Miltenyi, 130-059-901, Tokyo, Japan) in 1× PBS. After a 15 min incubation, the blocking reagent was replaced with an antibody solution (Table S1) at 1:100 each with 1 μg/mL of Hoechst 33,342 in 1× PBS. Panel 1 was used for PDOTS derived from primary tumor samples, and panel 2 was used for PDOTS derived from ascites samples. After a 15 min incubation, the medium channels were washed twice with 1× PBS before imaging using an inverted Nikon Eclipse Ti microscope equipped with Nikon DS-Qi1Mc camera and NIS-Elements software. Live–dead analysis was performed on separate samples to determine cell viability in each treatment condition following 5–7 days of treatment. A 1:1 solution of AO/PI stain (Nexcelcom, CS2-0106, Lawrence, MA, USA) in 1× PBS was made and 20–30 μL of this solution was added to each device after the medium was drained. After a 5 min incubation with the stain, the devices were imaged using a Nikon Eclipse Ti microscope. Using this software, the number of live cells stained with acridine orange (AO) and dead cells stained with propidium iodide (PI) were quantified for analysis.

### 2.7. Identification of Early Markers of Immune Activation in CD4+ and CD8+ T Cells

Patient-derived solid tumor samples were collected surgically and cut into approximately 2 cm^3^ pieces, avoiding any fatty or fibrotic material. 2 to 3 of these pieces were placed into a 15 mL centrifuge tube containing approximately 4 mL of warm digestion buffer composed of RPMI base medium supplemented with 10% FBS, 1% penicillin-streptomycin, 100 U/mL collagenase type IV (Gibco, 17104019), and 50 μg/mL Dnase I (Sigma-Aldrich, 04716728001). The tumor was minced with sterile scissors for approximately 2 min, after which the supernatant was transferred to a separate tube containing only RPMI with 10% FBS and 1% penicillin-streptomycin. The digestion medium was replaced, and this process was repeated about three times. The resulting cells were pelleted and resuspended in a 6-well ULA plate containing the RPMI medium supplemented with 10% FBS, 1% penicillin-streptomycin, and 100 U/mL IL-2. Separate wells were treated with the following conditions: control, 2 µM of 5-aza and 2 µM of HDACi, 200 μg/mL of αPD-1 and αPD-L1, and a combination of all therapies. The cells were left to incubate for 24 h.

### 2.8. Flow Cytometry of Immune Cells

In total, 1–2 mL of the S2 + S3 patient ascites fraction was centrifuged and trypsinized to dissociate tumor spheroids within the sample. After 5 min, the trypsin was quenched with medium, and the cells were pelleted. The cells were washed with 1× PBS and pelleted again. Afterwards, the samples were incubated in the dark with Zombie NIR viability dye (Biolegend, 423105, Tokyo, Japan) resuspended in 1× PBS for 15 min. The cells were then washed again with 1× PBS supplemented with 2% FBS (FACS Buffer) and pelleted. Blocking was done using a 1:100 FcR blocking reagent (Miltenyi, 130-059-901) in FACS buffer. After 15 min of blocking, the cells were stained with their respective antibodies (Table S2) and resuspended in FACS buffer for another 15 min. Panel 1 focused on T cells/PD-1 expression and consisted of antibodies for CD45, CD3, CD8, CD4, and PD-1. Panel 2 focused on PD-L1 expression and consisted of antibodies for CD45, EPCAM, and PD-L1. After staining, the cells were fixed with either 2% formaldehyde or 4% paraformaldehyde in the dark for 30 min, washed with 1.5 mL of FACS buffer, and pelleted. The cells were resuspended in 200 µL of the FACS buffer and analyzed using a BD LSR Fortessa. Data gating and analysis were done using the FlowJo 2 software. Gating descriptions can be found in Table S3. For the solid S1 patient sample, the above protocol was followed using the antibody panel listed in Table S4.

### 2.9. Assessment of Therapeutic Efficacy in Syngeneic HGSOC Models

The animal studies were reviewed and approved (#2016N000212) by the Institutional Animal Care and Use Committee (IACUC) of Brigham and Women’s Hospital. Tumor engraftment was completed through intraperitoneal injection on 6-week-old immunocompetent C57BL/6J female mice (Jackson Laboratories, Strain #000664, Bar Harbor, ME, USA) with 3.7 × 10^6^ KPCA.B cells resuspended in a 1:1 solution of 1× PBS and Matrigel (Corning, 354234). The mice were treated with intraperitoneal injection of 5-aza resuspended in 1× PBS at 4 mg/kg, HDACi resuspended in 1× PBS at 2 mg/kg (Fisher Scientific, 0000001045), 50 µg of αPD-L1 (Bio X Cell, BE0101, Lebanon, NH, USA) diluted in a 6.5 pH dilution buffer (Bio X Cell, IP0065), and 50 µg of αCTLA-4 (Bio X Cell, BE0131) diluted in a 7.0 pH dilution buffer (Bio X Cell, IP0070). A total of four conditions were utilized: control, 5-aza and HDACi treatments four times a week on alternating weeks, αPD-L1 and αCTLA-4 treatments twice a week, and 5-aza and HDACi treatments four times a week on alternating weeks, with αPD-L1 and αCTLA-4 treatments twice every week. Tumor burden was quantified by isolating and measuring all tumors at necropsy. Tumor engraftment was considered successful if the final tumor mass was greater than 50 µg. Mice with unsuccessfully engrafted tumors (below threshold) were excluded in the study. Only mice that survived past day 30 had their tumor masses quantified.

### 2.10. Statistical Analysis

Statistical analysis was completed for immunofluorescent imaging, cytokine analysis, and live–dead imaging of patient-derived organoid tumor devices using the Prism 9 software. Paired two directional t-tests were used for cytokine analysis and a one-way ANOVA, with post hoc Tukey tests, was used for all other statistical analysis. The results were deemed significant if *p* values were equal to or less than 0.05 (*p* ≤ 0.05). The results are shown as mean ± SD for in vitro studies and mean ± SEM for in vivo studies. The GeoMx DSP Data Analysis Suite (v2.4.0.421) was used to evaluate the raw count data output from the nCounter platform. As the counts for the three housekeeping control antibodies (Histone H3, GAPDH, and S6) were sufficiently high and concordant, we used the geometric mean of all three for normalization. For statistical testing, the negative controls (Ms IgG1, Ms IgG2a, and Rb IgG1) and the housekeeping genes (Histone H3 and GAPDH, S6) were removed, leaving 71 protein targets. Statistical significance was determined using t-tests with Benjamini–Hochberg correction for multiple testing.

## 3. Results

### 3.1. Digital Spatial Proteomic Profiling of Patient TILs in HGSOC

**Figure 1:**
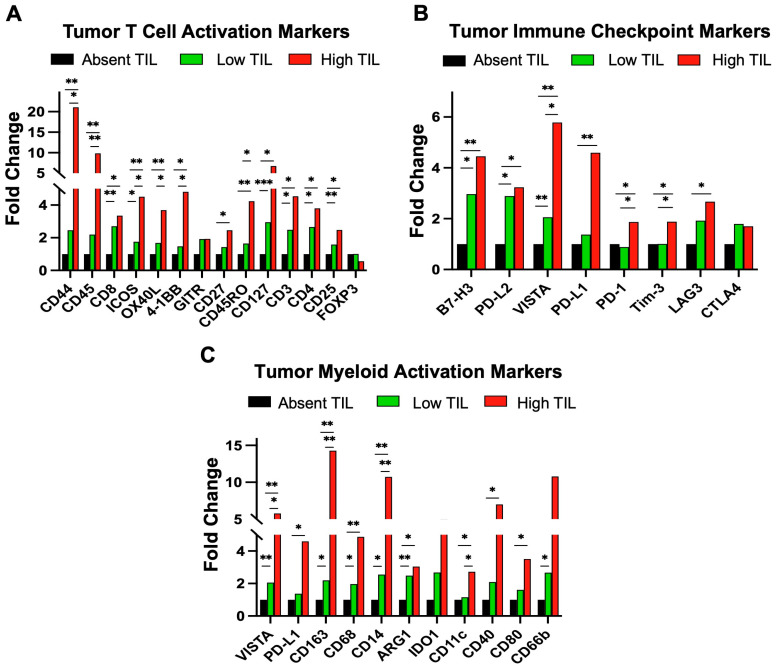
GeoMx digital spatial profiling. The signal from absent TIL was set as baseline. (**A**) GeoMx DSP proteomic analysis of tumor T cell activation markers; (**B**) tumor-immune checkpoint markers; (**C**) and tumor myeloid activation markers in patient samples with high TILs, low TILs, and minimal or absent TILs (* *p* ≤ 0.05; ** *p* ≤ 0.01; *** *p* ≤ 0.001).

Unlike melanoma, immunotherapeutic responses in HGSOC have been lower than expected and longer-term cures have been hard to achieve with ICB, with clinical trial responses seen in 10–15% of patients [46]. Furthermore, the hypoxic and acidic TME seen in HGSOC can co-opt myeloid cells to promote a pro-tumorigenic and immune suppressive phenotype while blocking immune cell proliferation, activation, and infiltration [46,47]. Three formalin-fixed paraffin embedded (FFPE) primary ovarian cancer patient samples were selected for digital spatial proteomic (DSP) analysis by a board-certified pathologist. From these 3 slides, 24 tumor and 16 stroma segments were identified and evaluated for changes in immune-related protein expression. These segments were categorized according to their level of infiltration: absent TILs, low TILs, and high TILs (Figure 1 and Figure S1).

To investigate the functionality of effector T cells in heavily pretreated patients and better understand the low response to ICB in HGSOC clinical trials, we analyzed high (brisk) TILs that infiltrate diffusely within the tumor and compared them to low (non-brisk) TILs that show only focal infiltration and absent TILs. We used the Nanostring GeoMx platform, which allows spatial digital (microregional) single-cell proteomic profiling using 77 antibodies on a FFPE slide. Using this platform, we saw specific upregulation of tumor T cell activation, immune checkpoint, and myeloid activation markers. DSP analysis showed an increase by fold change (FC) in almost all T cell activation-associated markers in tumor regions with high TILs compared to low or absent TILs (Figure 1A). We found that immunostimulatory markers, such as CD25 (*p* = 0.02), CD127 (*p* = 0.02), and CD27 (*p* = 0.01), were all upregulated in high TILs. As expected, the total number of leukocytes in high TILs was higher than in absent TILs (CD45; *p* = 0.002). Additionally, high TILs showed higher levels of total T cells (CD3; *p* = 0.02), cytotoxic T cells (CD8; *p* = 0.01), T helper cells (CD4; *p* = 0.01), and memory T cells (CD45RO). CD44, a cell adhesion molecule involved in effector-memory T cell activation and cell migration [48], showed the largest fold change increase in high TILs (*p* = 0.008). The results were tumor-specific and stroma effects were relatively minor, although some markers followed similar trends (Supplementary Figure S1). 

We also identified highly exhausted T cells with concurrent upregulation of multiple immune checkpoint markers in high TILs (Figure 1B). Upregulation of immune checkpoint markers characteristic of exhausted T cells was observed on high TILs, including PD-1, B7-H3, VISTA, Tim-3, and LAG3. B7-H3 inhibits tumor antigen-specific immune responses. Thus, brisk TILs showed increased expression of key immune checkpoint markers, such as VISTA (*p* = 0.003), PD-1 (*p* = 0.03), and B7-H3 (*p* = 0.001), which are associated with T cell exhaustion [49,50]. The functional severity of T cell exhaustion likely correlates with the number and magnitude of immune checkpoint protein expression, especially VISTA.

Myeloid cell activation-associated markers in high TILs followed the same trend as the T cell activation-associated markers (Figure 1C). Macrophage levels were found to be increased in high TILs (CD68; *p* = 0.01). These increases involved both M1-like macrophages (CD80; *p* = 0.01) and M2-like macrophages (CD163; *p* = 0.003). Interestingly, the largest increase involved immunosuppressive M2 macrophages (CD163). Upregulation of CD80, which has a crucial role in binding to CD28 and triggering activation of T cell immune function [51], was also noticed in high TILs. In addition, the upregulation of IDO1, which has immunosuppressive properties [52,53], was also present. Furthermore, neutrophils (CD66b) were elevated, and they have been correlated with worse progression-free survival and overall survival in other solid tumors [54,55]. Monocytes (CD14; *p* = 0.001), conventional dendritic cells (CD11c; *p* = 0.04), and antigen-presenting cells (CD40; *p* = 0.01) were all elevated in high TILs, with each being linked to either immunosuppression and/or tumor proliferation [56,57,58]. The low (non-brisk) TILs reflected these immune-associated differences, albeit to a lower extent, compared to non-TILs. Given the observed highly immunosuppressive tumor microenvironment and highly exhausted TILs, we sought to develop strategies that would enhance the potency of ICB immunotherapies, leading to the enhanced functionality, migration, and target engagement of T cells to tumor cells.

### 3.2. Epigenetic Priming Reverses Loss of 5-hmC and Mediates Upregulation of Key Tumor Cytokines 

**Figure 2:**
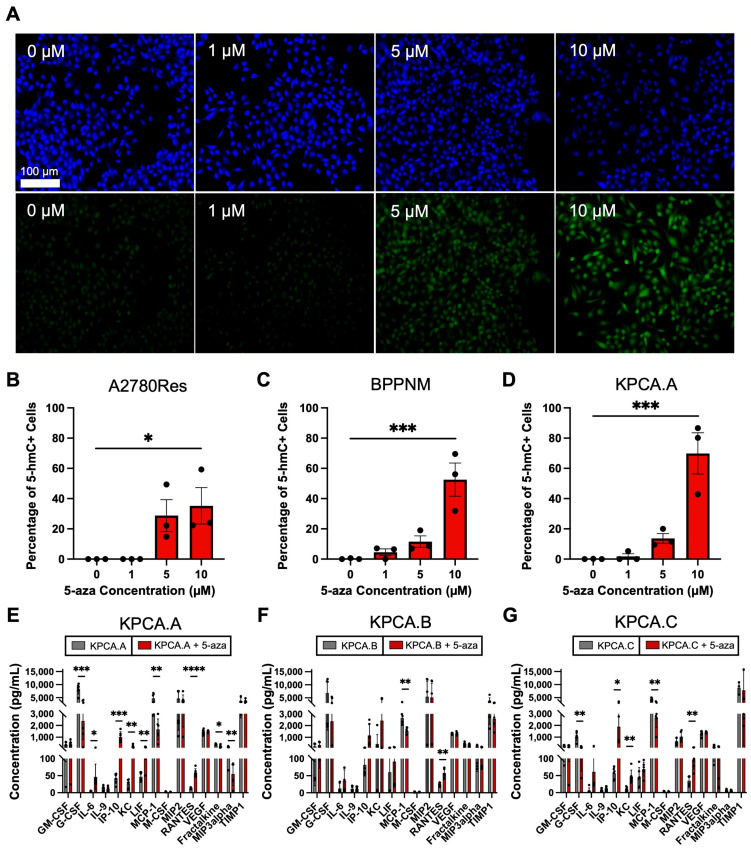
Effects of 5-azacytidine on tumor methylation and cytokine secretion. (**A**) Representative images of A2780Res human DAPI (**top**) and 5-hmC (**bottom**) immunofluorescent staining in response to increasing 5-aza concentrations. Scale bar, 100 μm; (**B**) Quantification of 5-hmC levels in response to 5-aza treatment for A2780Res; (**C**) BPPNM and (**D**) KPCA.A; (**E**) Quantification of cytokine expression with and without 5-aza treatment in KPCA.A; (**F**) KPCA.B and (**G**) KPCA.C (*n* = 6). (* *p* ≤ 0.05; ** *p* ≤ 0.01; *** *p* ≤ 0.001; **** *p* ≤ 0.0001).

We previously reported that epigenetic priming increases sensitivity to standard chemotherapies [38]. Given the low responses seen in ICB clinical trials in HGSOC and the key role of DNA methylation in modulating responsiveness to ICB [20,21,22,23,24,25,26,27,28,29,30,31,32,33,34,35,36,37], we investigated whether epigenetic priming could potentiate immunotherapies in this tumor subtype. Similar to our previous results [38], we first confirmed that DNMTIs, such as 5-aza, can be used to reverse global 5-hmC loss in HGSOC. We quantified 5-hmC expression in response to 5-aza treatment in multiple tumor lines using immunofluorescence studies. The ovarian cancer cell lines included human chemoresistant A2780Res and several murine syngeneic HGSOC tumor lines with fallopian tubal origin and key genetic alterations found in patients: BPPNM (*Brca1^−/−^*; *Trp53^−/R172H^*; *Pten^−/−^*; *Nf1^−/−^Myc^OE^*) and a series of KPCA (*Trp53^−/R172H^*; *Ccne1^OE^*; *Akt2^OE^*; *KRAS^G12V^*) tumor lines with varying degrees of immunotherapeutic resistance [39,41]. The KPCA cell line series consisted of a relatively responsive tumor line (KPCA.B), partially resistant (KPCA.A), and resistant (KPCA.C) tumor line to ICB therapy [59]. The percentage of 5-hmC positive cells showed a statistically significant increase in response to 10 µM 5-aza for all lines (Figure 2A–D and Figure S2), indicating that 5-aza treatment leads to an increase in global demethylation in tumor cells similar to our previous findings [39]. Specifically, the A2780Res human chemoresistant cell line showed a significant increase in the percentage of 5-hmC positive cells at a concentration of 10 µM (*p* ≤ 0.05) and the syngeneic murine HGSC BPPNM and KPCA.A lines exhibited similar trends at 10 µM with higher significance (*p* ≤ 0.001, both) (Figure 2B–D).

Next, we sought to investigate a potential mechanism of action for DNMTis to prime and enhance the ICB response. We thus treated the three KPCA cell lines (KPCA.A, KPCA.B, KPCA.C) for 72 h with 5-aza and observed changes in tumor-derived cytokine secretion (Figure 2E–G). Conditioned tumor cell medium treated with 10 μM 5-aza showed upregulation of 4 key cytokines—IL-6, IP-10 (CXCL10), KC (CXCL1), and RANTES (CCL5)—which activate both adaptive and innate immune responses (Figure 2E–G). IL-6 is known to recruit neutrophils and promote the differentiation of T and B cells [59]. KC (CXCL1) can function as a neutrophil chemoattractant [60]. IP-10 (CXCL10) is a well-known chemoattractant that increases the expression of interferon genes and promotes effector CD8+ and NK tumor infiltration [61]. RANTES (CCL5) functions as a chemoattractant for monocytes, T effectors, and NK cells [62,63]. Statistically significant upregulation was observed for all four key markers as follows: IL-6 (*p* ≤ 0.05, KPCA.A), IP-10 (*p* ≤ 0.001, KPCA.A; *p* ≤ 0.05, KPCA.C), KC (*p* ≤ 0.01, KPCA.A and KPCA.C), and RANTES (*p* ≤ 0.0001, KPCA.A; *p* ≤ 0.01, KPCA.B and KPCA.C). Conversely, significant downregulation was observed for cytokines that confer a pro-tumor effect, such as G-CSF (*p* ≤ 0.001, KPCA.A; *p* ≤ 0.01, KPCA.C), MCP-1 (*p* ≤ 0.01, all lines), and MIP3alpha (*p* ≤ 0.05, KPCA.A) [64,65,66]. 

### 3.3. Optimization of Patient-Derived 3D Ex Vivo Platforms for Rapid Immunotherapeutic Testing of HGSOC Tumors and Ascites 

**Figure 3:**
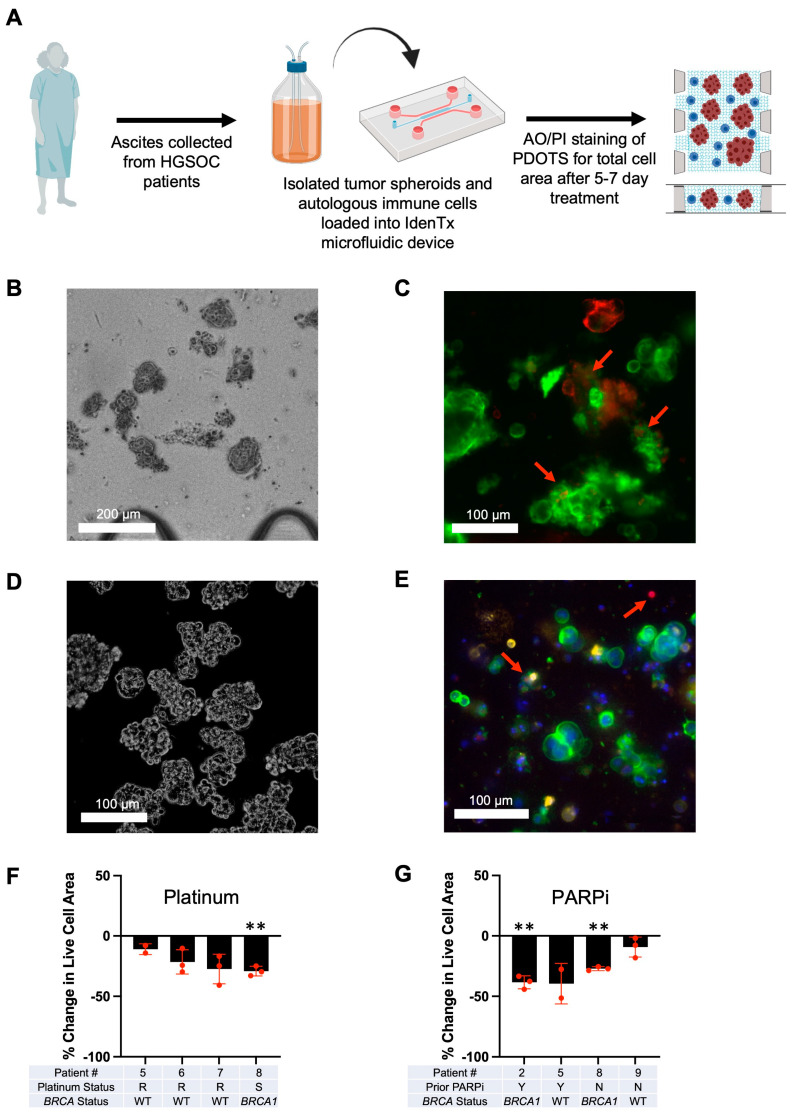
Scheme of PDOTS development, imaging of tumor spheroids, and clinically relevant treatments. (**A**) Scheme depicting how ascites samples were collected from ovarian cancer patients and processed to generate PDOTS; (**B**) Brightfield image of isolated primary tumor spheroids inside the IdenTx device. Scale bar, 200 μm; (**C**) Immunofluorescent image of PDOTS tumor spheroids from patient solid HGSC tumor stained with EpCAM (green) and CD45 (red). Red arrows indicate CD45+ immune cells. Scale bar, 100 μm; (**D**) Brightfield image of PDOTS tumor spheroids from patient ascites. Scale bar, 100 μm; (**E**) Immunofluorescent image of PDOTS tumor spheroids from patient HGSC tumor-derived ascites stained with Hoechst (blue), EpCAM (green), CD8 (red), and PD-L1 (gold). Red arrows indicate CD8+ immune cells. Scale bar, 100 μm; (**F**) Cumulative live–dead staining data of 10 µM of platinum treatment normalized to control of each patient; (**G**) Cumulative live–dead staining data of 1 µM of PARPi treatment normalized to control of each patient (** *p* ≤ 0.01).

Tumor spheroids, along with autologous immune cells, were isolated from patient ascites and processed via a standardized methodology (Figure 3A) to rapidly examine therapeutic combinations ex vivo. First, a primary tumor sample was loaded into the IdenTx device (Figure 3B) to demonstrate the standard morphology of a PDOTS sample. Subsequent immunofluorescent imaging (Figure 3C) showed that EpCAM-positive tumor cells were present in addition to regions of embedded CD45-positive immune cells. These findings were emulated with cells derived from patient ascites, as seen in images of the PDOTS in Figure 3D. This patient sample also demonstrated tumor spheroids, as seen by the EpCAM positivity and CD8 positive immune cells (Figure 3E). There also appear to be regions of PD-L1 positivity on the EpCAM tumor cells that are accessible to target with ICB immunotherapies (Figure 3E).

On selected patient ascites samples, we used standard-of-care therapies, such as platinum-based and PARPi therapy, to further validate our ex vivo platform. As seen in Figure 3F, when the devices were treated with 10 µM cisplatin, the only patient to show significant cell death compared to their control was patient 8, who was clinically diagnosed as platinum sensitive (Table S5). When the sample from patient 7 was first tested, the clinic informed our team that the patient was platinum sensitive. However, when cisplatin was tested on their sample, there was a reduced response. Upon following up with the clinic, we were told that the patient’s chart was updated to indicate that the disease had become platinum resistant, which validates the sensitivity of our device. Patients with known *BRCA* mutations (Table S5) showed significant responses to PARPi (patients 2 and 8), while patient 5, with no known *BRCA* mutation, showed no significant response to PARPi (Figure 3G).

### 3.4. Epigenetic Priming Potentiates ICB in PDOTS and Upregulates Early Activation Immune T Cell Markers

**Figure 4:**
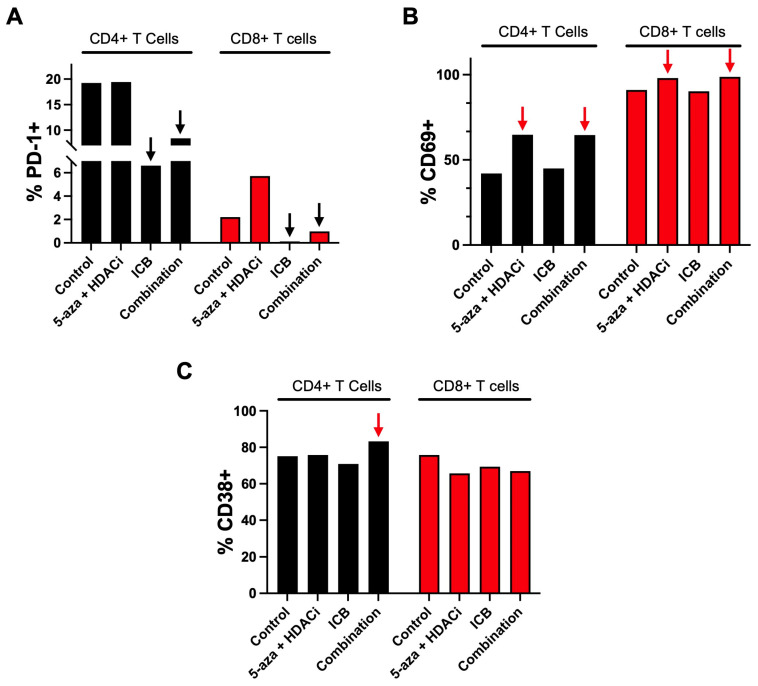
Immune profiling of solid HGSOC patient sample. After 24 h of treatment, markers for immune activation were quantified. Black arrows indicate downregulation and red arrows indicate upregulation. (**A**) Percentage of CD4+ and CD8+ T cells expressing PD-1; (**B**) Percentage of CD4+ and CD8+ T cells expressing CD69; (**C**) Percentage of CD4+ and CD8+ T cells expressing CD38.

A surgically resected primary HGSOC tumor was dissociated into PDOTS and treated for 24 h with 5-aza, HDACi and immunotherapies to observe the activation of immune cells. CD4+ and CD8+ T cells both had an observable downregulation of PD-1 (Figure 4A) in conditions treated with immunotherapies. We attribute the downregulation to the αPD-1 immunotherapy blocking the conjugated flow cytometry antibody from binding to the epitope, thus validating the treatment. Additionally, both CD4+ and CD8+ T cells treated with our 5-aza + HDACi treatment or the combination treatment had an upregulation of CD69 (Figure 4B), which is an early immune activation marker [67,68]. Another early immune activation marker, CD38 [69], was upregulated in CD4+ T cells when treated with the combination therapy (Figure 4C).

**Figure 5:**
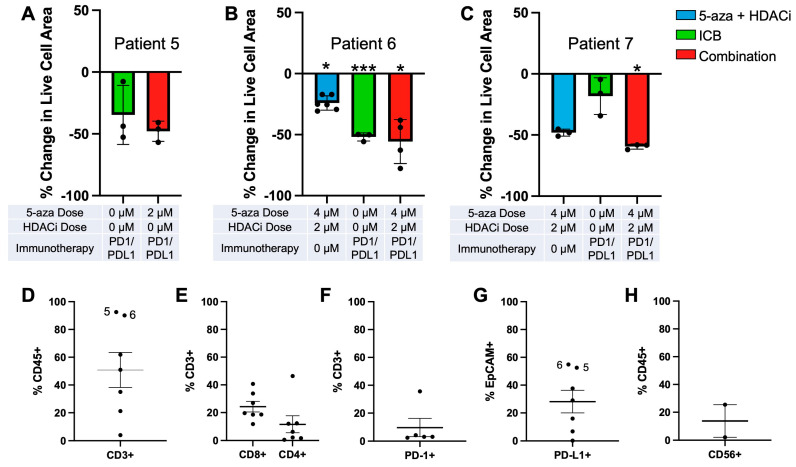
PDOTS utilizing circulating tumor from patient ascites and immune profiling. Cumulative live–dead staining data normalized to control. Significance is shown in comparison to each individual sample’s control condition. (**A**) Patient 5; (**B**) Patient 6; (**C**) Patient 7; (**D**) CD3+ expression as a percentage of total CD45+ cells (*n* = 7). Patient sample 5 and 6 percentages indicated; (**E**) CD8+ and CD4+ expression as a percentage of total CD3+ cells (*n* = 7); (**F**) PD-1+ expression as a percentage of total CD3+ cells (*n* = 5); (**G**) PD-L1+ expression as a percentage of total EpCAM+ cells (*n* = 7); Patient sample 5 and 6 percentages indicated; (**H**) CD56+ expression as a percentage of total CD45+ cells (*n* = 2). (* *p* ≤ 0.05; *** *p* ≤ 0.001).

Various combinations of 5-aza, HDACi, and immunotherapies were tested on ascites PDOTS to find a synergistic treatment that repeatedly gave significant results in the combination treatment. Response to treatments was measured by taking the area of live cells and dividing it by the total area of both live and dead cells (Figure S3A). Significance is determined by comparing it to the control. Of the seven tested patient ascites samples, 3/7 responded to treatment with epigenetic modulators, 1/7 responded to treatment with immunotherapies, and 4/7 responded to a combination of treatments (Figure 5A–C and Figure S3B–E). The combination treatment indicates a trend whereby priming with epigenetic modulators can increase the effectiveness of immunotherapy treatments.

Of note, patient 5 (Figure 5A) did not show a significant response to αPD-1 and αPD-L1 immunotherapies alone, but the combination treatment did approach significance with a *p*-value of 0.06, showing a trend towards treatment synergism. Of the patient ascites samples, patient 6 (Figure 5B) was the only sample that showed significant cell death when treated with αPD-1 and αPD-L1 immunotherapies alone. Patient 7 (Figure 5C) was the only patient sample that responded to only the combination treatment and neither the epigenetic nor immunotherapy-alone conditions. Additionally, Kuramochi cells expressing the necessary HLA protein (Figure S4A) for non-autologous immune activation [70] also showed a significant response to the combination treatment when cocultured with T cells in the IdenTx devices (Figure S4B).

Flow cytometric analysis of the patient ascites samples (Figure 5D–H) showed large variability in fluid composition. CD3+ T cells made up about 50% of CD45+ immune cells (Figure 5D). Of these T cells, roughly 25% were CD8+ cytotoxic T cells and roughly 10% were CD4+ helper T cells (Figure 5E). Additionally, about 10% of CD3+ T cells were also PD-1+ (Figure 5F). EPCAM was utilized as a marker for circulating tumor cells, and roughly 30% of EPCAM+ cells were also PD-L1+ (Figure 5G). Notably, the only responders to the immunotherapy-alone condition (patient 5 and patient 6) correlated with an increased percentage of total CD3+ T cell populations (Figure 5D) and PD-L1 expression in EPCAM+ circulating tumor cells (Figure 5G).

### 3.5. Epigenetic Priming Increases Overall Survival by Potentiating ICB in Syngeneic HGSOC Models

**Figure 6:**
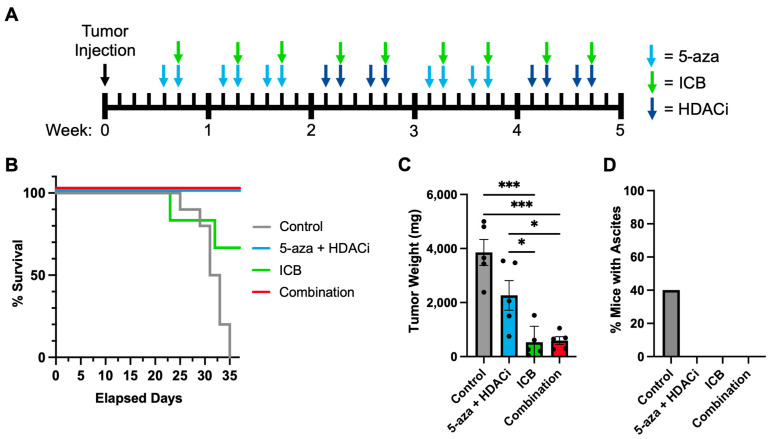
In vivo mouse model engrafted with KPCA.B cells. (**A**) Treatment schedule; (**B**) Survival curve of control (*n* = 10), 5-aza + HDACi (*n* = 5), ICB (*n* = 6), and combination (*n* = 5); (**C**) Quantification of tumor weights at sacrifice day (day 35) for the control cohort (*n* = 5), 5-aza/HDACi (*n* = 5), ICB (*n* = 5), and 5-aza/HDACi/ICB combination (*n* = 5); (**D**) Percent of mice that developed ascites in the controls at day 35 (*n* = 5), 5-aza + HDACi (*n* = 5), ICB (*n* = 5), and combination (*n* = 5) conditions. (* *p* ≤ 0.05; *** *p* ≤ 0.001).

In vivo experimentation was conducted with KPCA.B cells to identify a potential benefit in combining 5-aza, givinostat (HDACi), and ICB (PD-L1/CTLA-4) in syngeneic HGSOC tumor models that recapitulate the clinical disease and ICB responses [41]. The experimental design is outlined in Figure 6A. Overall, our results recapitulated previous tumor model studies [40], although we saw more subtle effects likely due to testing epigenetic priming in a tumor model relatively sensitive to ICB therapy. Most importantly, epigenetic priming increased overall survival, as all mice treated with combination therapy were alive at the end of the experiment (5/5) compared to immunotherapy-treated mice (4/6) and controls (5/10) (Figure 6B). The survival results of the control cohort were similar to those reported by Iyer et al. [41], who described the development of syngeneic HGSOC models. All remaining animals were sacrificed on day 35, when tumor burden endpoints were reached in the control cohort (Figure 6C). The tumor weights of all sacrificed mice are quantified in Figure 6C. The mice that were treated with 5-aza/HDACi/ICB combination had significantly smaller tumor masses than the control group (*p* = 0.0001) and the 5-aza/HDACi cohort (*p* = 0.0381) (Figure 6C). The control mice that were left untreated developed ascites consistent with previous animal models [40], while the other groups did not present ascites (Figure 6D). Overall, the combination-treated mice were a homogenous cohort in which all mice responded to ICB, had better overall survival, and had significantly lower tumor weights compared to the control and epigenetic therapy cohorts. The ICB-treated group was a more heterogenous group composed of both ICB responders and non-responders. Several mice that did not respond to ICB followed a survival pattern consistent with control mice and died before the 35-day mark. Mice that survived responded well and had low tumor weights comparable to those seen in combination-treated mice. These results, at least in this syngeneic tumor model, are more nuanced than the conclusions reached by previous ID8 studies [40] and appear to suggest that epigenetic priming likely confers additional benefit primarily to weak responders or non-responders to ICB therapy. 

## 4. Discussion

The main goal of our study is to develop 3D IO platforms to analyze patient responses and identify adjuvants, such as epigenetic drugs, that are able to potentiate systemic immunotherapy responses in tumor ascites. Towards this goal, we developed rapid, sensitive, and accurate functional systems based on ovarian patient-derived 3D PDOTS and expanded their use beyond solid tumors to study patient ascites. This allows us to study the complex and dynamic tumor-immune interactions in the systemic circulation and identify personalized profiles associated with the engagement of circulating tumor and immune cells that are primed for response to ICB immunotherapies. This patient-derived 3D platform fulfills a critical need for clinical tools that can accurately assess drug sensitivity and treatment responses in real time. While most investigations studying response to immunotherapies in ovarian cancer focus on characterizing biomarkers and immune cell infiltration in primary solid tumors, we instead concentrated on developing PDOTS containing both circulating ascites tumors and immune cells collected from the same patients in the setting of relapsed HGSC disease (either with pathogenic *BRCA1*, *BRCA2* mutations, or *BRCA* WT). 

Most importantly, systemic responses are indicative of inefficient ICB responses seen in clinical trials. Moreover, we are showing that the 3D platform is predictive of clinical responses, as it accurately recapitulates responses to both platinum chemotherapy and PARPi responses in HGSC. Primary solid tumors are not relevant for ICB responses in ovarian cancer, since they are removed at diagnosis through surgical debulking. The main goal of ICB therapy in newly diagnosed HGSC patients following debulking or relapsed patients is to eliminate circulating tumor cells, which give rise to peritoneal metastases and contribute to disease progression. Consequently, this paper is novel and highly clinically relevant, as it allows the testing of readily accessible samples when solid tumors are not easily available or resectable. This includes liquid biopsies and tumor ascites through non-invasive methods of collection or as part of standard patient care.

A major aim of the current study was to identify biomarkers responsible for immune evasion in HGSOC, which could explain the low responses seen in ICB clinical trials for this tumor subtype. We employed digital spatial tumor profiling to investigate the functionality of effector T cells. Specifically, we analyzed TILs that infiltrate diffusively within the tumor and compared their functionality to non-brisk TILs that show only focal infiltration and also to non-TILs (no TILs or minimal TIL infiltration). Immunostimulatory markers, such as CD25 and CD27, were all upregulated in the brisk TIL sample. Additionally, the brisk TIL patient sample also showed significantly higher levels of total T cells (CD3), cytotoxic T cells (CD8), T helper cells (CD4), and memory T cells (CD45RO). CD44, a key activation marker for effector and memory T cells, showed the largest fold change increase between the brisk TIL sample and the absent TIL sample. CD45RO, which is expressed by memory T cells that have encountered antigens, was also upregulated in TILs. However, what we clearly saw was the presence of highly exhausted T cells and the upregulation of immune checkpoint markers. Upregulation of all immune checkpoint markers characteristic of highly exhausted T cells was seen on brisk TILs, including PD-1, B7-H3, VISTA, Tim-3, and LAG3. B7-H3 inhibits tumor antigen-specific immune responses. The functional severity of T cell exhaustion correlates with the number and magnitude of immune checkpoint protein expression. Most importantly, one of the biggest contributors to immune evasion is the presence of immunosuppressive M2-like macrophages, which show the highest elevation among myeloid immune cell markers. This suggests that therapies that trigger tumor-associated macrophage reprogramming from M2-like to M1-like phenotypes are likely to increase responsiveness to IO compounds. In addition, our results indicate that although TILs are present within the local immune microenvironment, they are highly exhausted and inefficient in detecting and destroying tumor cells located in their proximity. Consequently, epigenetic adjuvants, which induce demethylation of key sites, including PD1/PD-L1, could both reactivate and re-energize local and systemic immune responses. We thus suggest that combination treatments boosted by epigenetic priming could work synergistically to potentiate IO responses and more effectively target and eliminate tumor cells. 

Immunofluorescence staining analysis for 5-hmC levels was performed to determine the extent of global methylation levels in response to 5-aza treatment. Similar to our previous studies [39], 5-hmC positive cells were observed to increase in response to 10 µM 5-aza; 5-aza-mediated global demethylation allows for the activation of genes targeted by immunotherapy, such as PD1/PD-L1. In turn, ICB can promote anti-tumor immunity [49]. As such, combining immunotherapy and 5-azacytadine administration can potentially have synergistic effects in the treatment of ovarian cancer. Epigenetic priming induces secretion of key cytokines that are involved in activation of innate and adaptive immunity. Thus, significant upregulation was observed for multiple key cytokines, specifically IL-6, IP-10, KC, and RANTES, in the KPCA HGSOC tumor lines in response to 5-aza. In addition, a significant downregulation was observed for G-CSF, MCP-1, and MIP3alpha cytokines following 5-aza treatment. Interestingly, the cytokines that were upregulated are those associated with increased immune activation [59,60,61,63], while those downregulated are associated with cell migration and metastatic phenotypes [64,65,66]. IL-6 is a proinflammatory cytokine and plays an important role in the immune response by recruiting neutrophils and promoting the growth and differentiation of T and B lymphocytes [59,60,61,63]. IP-10 functions as a chemoattractant for monocytes, macrophages, T cells, NK cells, and dendritic cells under physiological conditions. Several studies have suggested that IP-10 contributes to anti-tumor immunity. IP-10 promotes infiltration by CD8+ tumor-infiltrating lymphocytes as well as increasing the expression of interferon genes [59,60,61,63]. Several studies have shown increased expression of IP-10 in ovarian cancer cells following treatment with DNMTi, consistent with our results [59,60,61,63]. Stone et al. observed significant regulation of IP-10 by 5-aza both in vitro and in vivo in syngeneic ID8 tumor models as well [40]. Similarly, RANTES is involved in immune and inflammatory responses and it is a potent chemoattractant for monocytes, T effector, NK, and dendritic cells [59,60,61,63]. Interestingly, epigenetic priming has also been shown not only to activate effector T, helper T, and NK cells but also to decrease the percentage of immunosuppressive myeloid derived suppressor cells (MDSCs) and M2-like macrophages in the tumor microenvironment [40].

Consequently, we found that epigenetic priming potentiated ICB in patient-derived 3D ex vivo platforms and syngeneic tumor models, which recapitulated the HGSOC cell of origin and clinical disease [40]. The combination of epigenetic priming and IO yields the best overall responses and the highest overall survival in vivo. Of note, the tumor microenvironment of the HGSOC tumor models evaluated in the current study is heterogenous, similar to patients, which results in variable IO responses. The results of our in vivo study show that the IO non-responsive murine group derives the greatest benefit in response to epigenetic tumor priming. The ex vivo patient data are also in alignment with this conclusion. The analysis of patient samples indicates that epigenetic priming potentiates IO responses overall and especially in IO-resistant disease, which remains a critical priority clinically.

In addition, our studies indicate that ex vivo-based PDOTS platforms have the potential to bridge the gap between animal studies and clinical trials. When utilizing standard care treatments, such as platinum and PARPi, we found results consistent with those seen clinically in patients, which increases the validity and predictive ability of our platform. An increased response to anti-PD-1/anti-PD-L1 ICB alone correlated with a high frequency of CD3+ T cells and PD-L1+/EPCAM+ tumor cells and was seen in a small number of patients. A combination of epigenetic adjuvants and ICB drastically enhanced the potency of ICB therapeutics, with effective responses seen in the majority of patients. Interestingly, epigenetic priming alone and in combination with ICB immunotherapy in patient-derived PDOTS induced the rapid upregulation of CD69, a reliable early activation marker in both CD4+ and CD8+ T cells [67,68]. Furthermore, downregulation of anti-PD-1 was seen in both ICB alone and combination treatments, validating the target responses. Consequently, this functional precision medicine approach has the ability to rapidly identify specific combinations able to potentiate ICB, which is a great advantage, especially as we consider the current clinical difficulty of fast and accurate testing of a high number of potential combinations directly in patients. In addition, it could identify ineffective combinations and spare patients from deleterious toxic side effects. Recent findings by Huang et al. [38] synergize with our results by indicating that PD-L1 demethylation is key in potentiating the PD-1/PD-L1 interaction and enhancing tumor immunosurveillance. Conversely, PD-L1 hypermethylation, especially at the K162 site, critically inhibits the PD-L1/PD-1 interaction and is a negative predictive biomarker of ICB response in patients [38]. Furthermore, PD-L1 K162 methylation is abrogated in response to IL6 [38], which is the cytokine that we have shown to be upregulated in response to epigenetic priming of tumors. Therefore, it is highly likely that 5-aza promotes demethylation of PD-L1, including at the K162 site, enhances the PD-L1/PD-1 interaction, and potentiates ICB responses as a result.

Thus, by testing novel cancer immunotherapeutics in predictive ex vivo platforms using patient solid tumors or ascites, researchers can identify whether effective treatments transfer from murine models to human applications in a cost-effective and time-efficient manner. In recent years, the value of ascites for research has been recognized more and more [71,72]. The current findings are clinically relevant, as testing in PDOTS platforms could rapidly evaluate the most effective personalized immunotherapeutic options using solid tumors or circulating tumor cells in tumor-derived ascites. Identifying reliable response markers and assays, which could accurately predict response to immunotherapy and clinical outcome, is critical since functional ex vivo testing of individual patient samples will eliminate drugs to which the tumors are resistant, thus sparing patients unnecessary toxicity from ineffective treatment. In addition, it could allow the implementation of epigenetic reprogramming strategies capable of sensitizing immunoresistant tumors prior to or in conjunction with the delivery of ICB immunotherapies.

## 5. Conclusions

In this study, we sought to identify therapeutic strategies to potentiate immune activation and function. DSP analysis of patient tumor samples with varying levels of TILs showed an increased prevalence of immunostimulatory markers in high TIL samples. However, DSP also identified highly exhausted T cells with concurrent upregulation of multiple immune checkpoint markers in brisk TILs, including B7-H3, VISTA, Tim-3, LAG3, and PD-1, which could explain the low HGSOC responses seen in αPD-1 clinical trials. Additionally, we optimized a novel ascites-based ex vivo profiling assay using microfluidic devices that can be used to study liquid or circulating tumor cells in addition to solid tumors. Taken together, the results obtained in syngeneic HGSOC in vivo tumor studies and 3D PDOTS screening suggest that epigenetic adjuvants could potentiate the efficacy of ICB immunotherapies. The implementation of PDOTS platforms, in particular, will allow for a fast and effective method to screen drug combinations on solid tumors and ascites-derived patient samples with autologous immune cells. This functional precision medicine approach has the potential for wider research applications to interrogate local, systemic, and peripheral tumor immunity, since they all contribute to effective and durable IO responses. Furthermore, it will allow the identification of specific combinations that can reverse innate and acquired ICB resistance, which is a great advantage, especially as we consider the current difficulty of rapidly testing a high number of combinations in clinical trials.

## Data Availability

Data can be accessed from corresponding author upon request.

## Supplementary Materials

The following supporting information can be downloaded at: https://www.mdpi.com/article/10.3390/cancers15164128/s1, Table S1. Antibodies used for IF imaging of PDOTS. Table S2. Antibodies used for Flow Cytometry of Patient Ascites. Table S3. Gating used to Observe Cell Types or Protein Expression for Flow Cytometry of Patient Ascites. Table S4. Antibodies used for Flow Cytometry of Solid Patient Sample for Immune Activation. Table S5. Patient Data for Ascites Sample. Figure S1. GeoMx digital spatial profiling. Signal from absent TIL set as baseline. (A) GeoMx DSP proteomic analysis of stromal T cell activation markers, (B) stromal immune checkpoint markers, (C) and stromal myeloid activation markers. (*, *p* ≤ 0.05; **, *p* ≤ 0.01; ***, *p* ≤ 0.001; ****, *p* ≤ 0.0001). Figure S2. 5-hmC expression in tumor lines following 5-aza treatment. Representative images of human DAPI (top) and 5-hmC (bottom) immunofluorescent staining in response to increasing 5-aza concentrations in (A) BPPNM and (B) KPCA.A cell lines. Scale bar, 100 μm. Figure S3. Representative and cumulative live-dead results of patient ascites PDOTS and associated immune quantifications. (A) Images used from patient 1 to calculate live percent cell area. Live cells stained with acridine orange (green) and dead cells stained with propidium iodide (red). Cumulative live-dead staining data normalized to control with significance shown in comparison to each individual sample’s control condition of (B) Patient 1, (C) Patient 2, (D) Patient 3, (E) and Patient 4. (F) Composition of CD45+ immune populations in patient derived ascites. (*, *p* ≤ 0.05; **, *p* ≤ 0.01). Figure S4. PDOTS utilizing co-culture of Kuramochi tumor line and T cells harvested from PBMCs. (A) HLA composition of Kuramochi tumor line. (B) Cumulative live-dead staining data normalized to control. Significance is shown in comparison to sample’s control condition. (*, *p* ≤ 0.05).
