# Supplementary material for: Development of a Patient-Derived 3D Immuno-Oncology Platform to Potentiate Immunotherapy Responses in Ascites-Derived Circulating Tumor Cells
Source: Cancers (Basel). 2023 Aug 16;15(16):4128. doi: 10.3390/cancers15164128 (PMC10452550; doi:10.3390/cancers15164128)
Supplement: Supplementary file 1 [file cancers-15-04128-s001.zip › Table S1 - Patient-derived 3D Immuno-Oncology Platform.pdf]

Table S1. Antibodies used for IF Imaging of PDOTS

Antibody	Fluorophore	Supplier	Catalog	Panel	Concentration
CD45	AF488	BioLegend	304017	1	1:100
EpCAM	PE	BioLegend	357423	1 & 2	1:100
CD8	AF488	BioLegend	301024	2	1:100
PD-L1	APC	BioLegend	374513	2	1:100
