# Supplementary material for: Development of a Patient-Derived 3D Immuno-Oncology Platform to Potentiate Immunotherapy Responses in Ascites-Derived Circulating Tumor Cells
Source: Cancers (Basel). 2023 Aug 16;15(16):4128. doi: 10.3390/cancers15164128 (PMC10452550; doi:10.3390/cancers15164128)
Supplement: Supplementary file 1 [file cancers-15-04128-s001.zip › Table S2 - Patient-derived 3D Immuno-Oncology Platform.pdf]

Table S2. Antibodies used for Flow Cytometry of Patient Ascites.

Antibody	Fluorophore	Supplier	Catalog	Panel	Concentration
CD3	PE	BD Biosciences	552127	1	1:200
CD4	BV421	BioLegend	357423	1	1:200
CD8	AF488	BioLegend	301024	1	1:200
PD1	AF700	BioLegend	329951	1	1:200
CD45	APC	BioLegend	368511	1 & 2	1:200
EpCAM	FITC	BioLegend	324204	2	1:200
PDL1	PE-Cy7	BioLegend	329717	2	1:200
