# Supplementary material for: Development of a Patient-Derived 3D Immuno-Oncology Platform to Potentiate Immunotherapy Responses in Ascites-Derived Circulating Tumor Cells
Source: Cancers (Basel). 2023 Aug 16;15(16):4128. doi: 10.3390/cancers15164128 (PMC10452550; doi:10.3390/cancers15164128)
Supplement: Supplementary file 1 [file cancers-15-04128-s001.zip › Table S3 - Patient-derived 3D Immuno-Oncology Platform.pdf]

Table S3. Gating used to Observe Cell Types or Protein Expression for Flow Cytometry of Patient Ascites.

Desired Expression	Gates
T cells	CD3+ / CD45+
Helper T cell lymphocytes of total T cells	(CD4+ / CD3+) ÷ (CD3+ / CD45+)
Cytotoxic T cell lymphocytes of total T cells	(CD8+ / CD3+) ÷ (CD3+ / CD45+)
PD1	PD1+ / CD3+
PDL1	PDL1+ / EpCAM+
