# Supplementary material for: Development of a Patient-Derived 3D Immuno-Oncology Platform to Potentiate Immunotherapy Responses in Ascites-Derived Circulating Tumor Cells
Source: Cancers (Basel). 2023 Aug 16;15(16):4128. doi: 10.3390/cancers15164128 (PMC10452550; doi:10.3390/cancers15164128)
Supplement: Supplementary file 1 [file cancers-15-04128-s001.zip › Table S4 - Patient-derived 3D Immuno-Oncology Platform.pdf]

Table S4. Antibodies used for Flow Cytometry of Solid Patient Sample for Immune Activation.

Antibody	Fluorophore	Supplier	Catalog	Concentration
CD45	BV711	BioLegend	304050	1:200
CD3	AF700	BioLegend	300424	1:200
CD4	BV785	BioLegend	300554	1:200
CD8	PerCP Cy5.5	Thermo Fisher Scientific	BDB560662	1:200
TIM-3	APC	BioLegend	345012	1:200
PD-1	BV421	BioLegend	329920	1:200
CD69	FITC	BioLegend	310904	1:200
CD38	PE	BioLegend	303506	1:200
CTLA-4	BV605	BioLegend	369609	1:200
LAG-3	PE-Cy7	BioLegend	369309	1:200
