# Supplementary material for: Development of a Patient-Derived 3D Immuno-Oncology Platform to Potentiate Immunotherapy Responses in Ascites-Derived Circulating Tumor Cells
Source: Cancers (Basel). 2023 Aug 16;15(16):4128. doi: 10.3390/cancers15164128 (PMC10452550; doi:10.3390/cancers15164128)
Supplement: Supplementary file 1 [file cancers-15-04128-s001.zip › Table S5 - Patient-derived 3D Immuno-Oncology Platform.pdf]

Table S5. Patient Data for Ascites Samples

**Patient Number**	**Platinum Status**	***BRCA* Status**	**Prior PARP**
1	Refractory	Wild-Type	Untreated
2	Resistant	*BRCA1* Mutation	Treated
3	Resistant	Wild-Type	Treated
4	Refractory	Wild-Type	Untreated
5	Resistant	Wild-Type	Treated
6	Resistant	Wild-Type	Treated
7	Resistant	Wild-Type	Untreated
8	Sensitive	*BRCA1* Mutation	Untreated
9	Resistant	Wild-Type	Untreated
