# Supplementary figures and images for: Development of a Patient-Derived 3D Immuno-Oncology Platform to Potentiate Immunotherapy Responses in Ascites-Derived Circulating Tumor Cells
Source: Cancers (Basel). 2023 Aug 16;15(16):4128. doi: 10.3390/cancers15164128 (PMC10452550; doi:10.3390/cancers15164128)

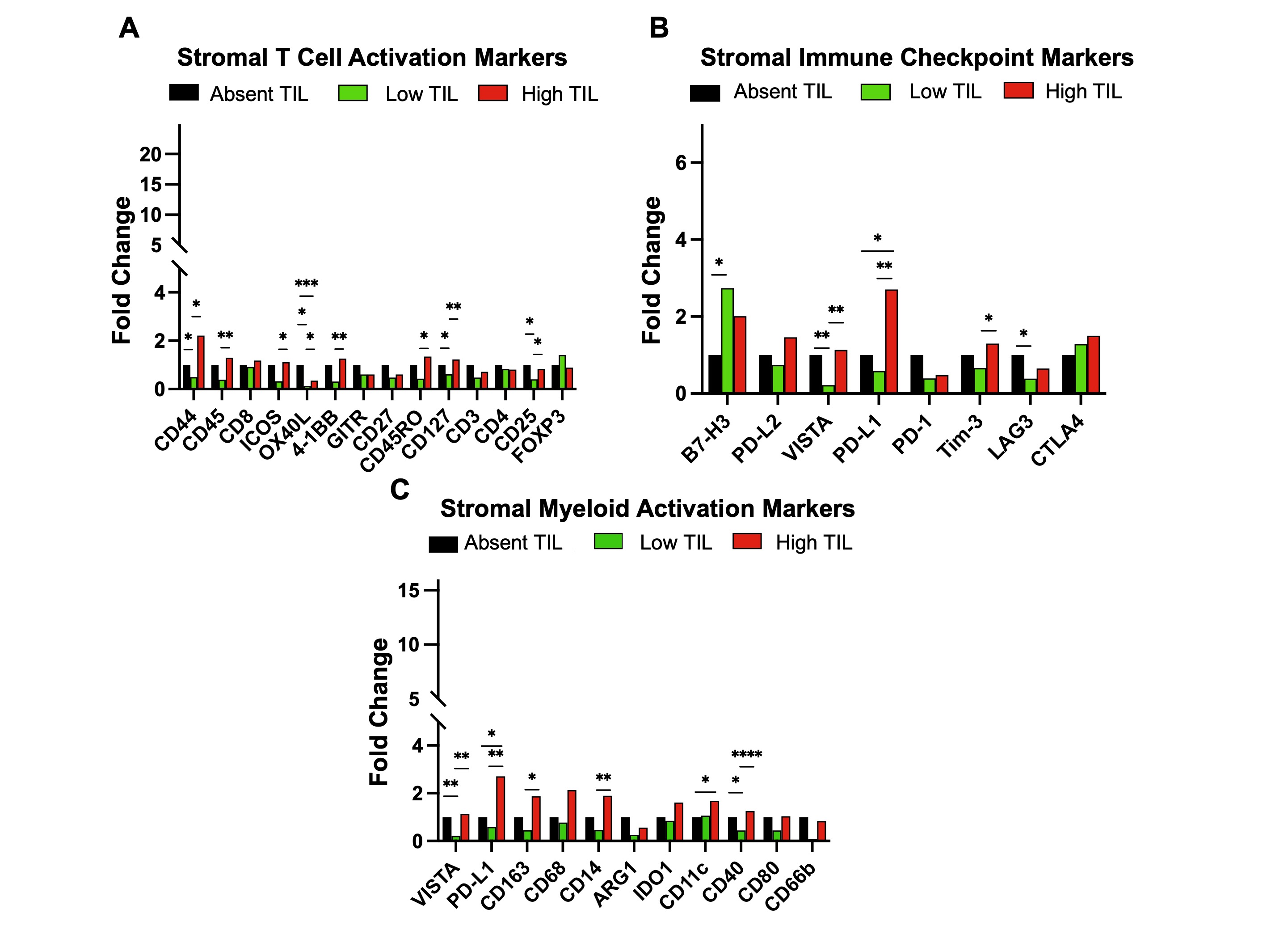

Supplement: Supplementary file 1 [file cancers-15-04128-s001.zip › Figure S1 - Patient-derived 3D Immuno-Oncology Platform.jpg]

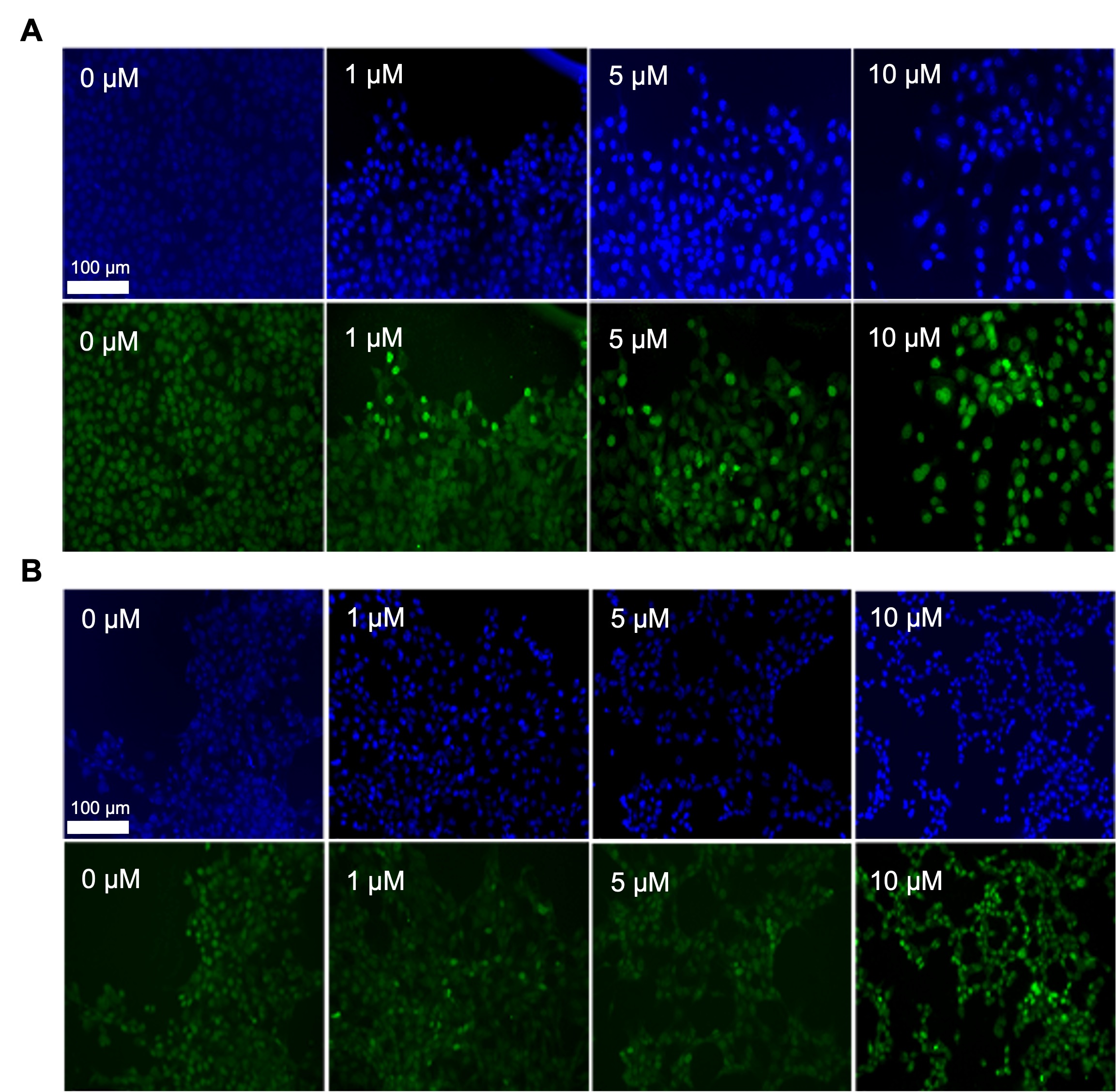

Supplement: Supplementary file 1 [file cancers-15-04128-s001.zip › Figure S2 - Patient-derived 3D Immuno-Oncology Platform.jpg]

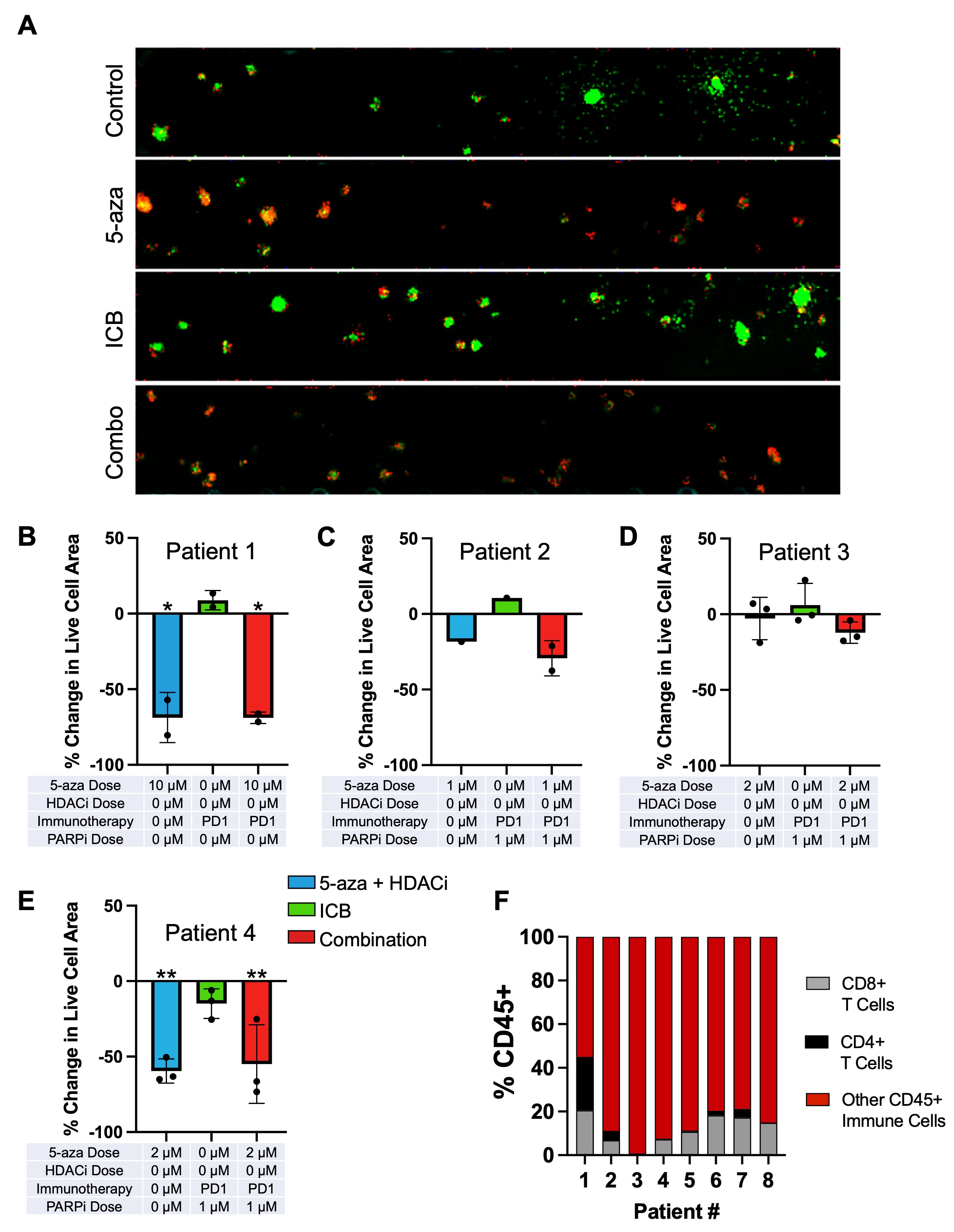

Supplement: Supplementary file 1 [file cancers-15-04128-s001.zip › Figure S3 - Patient-derived 3D Immuno-Oncology Platform.jpg]

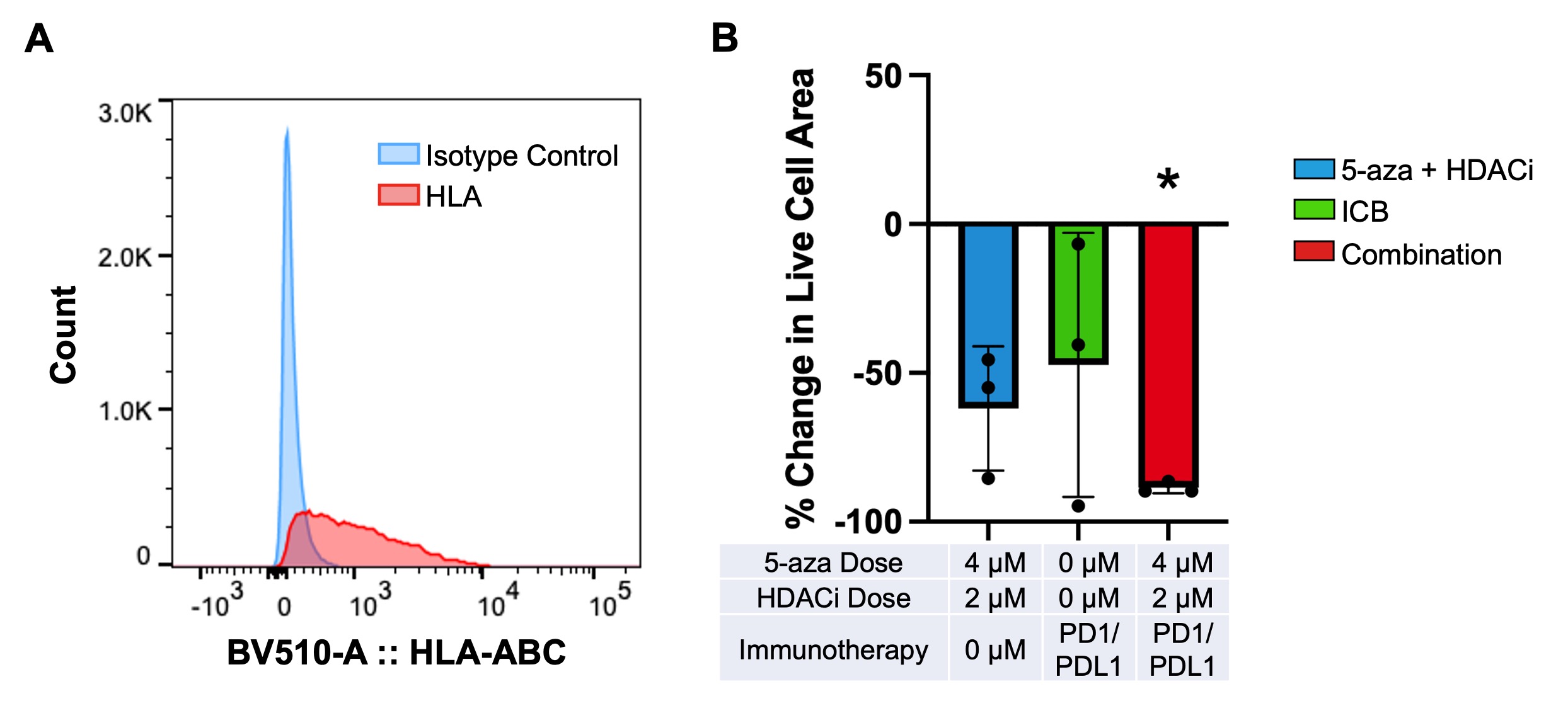

Supplement: Supplementary file 1 [file cancers-15-04128-s001.zip › Figure S4 - Patient-derived 3D Immuno-Oncology Platform.jpg]
